# Fever and temporal headache in a 70-year-old male with presumed large vessels vasculitis

**DOI:** 10.31138/mjr.31.2.220

**Published:** 2019-10-30

**Authors:** Christos Vettas, Athina Dimosiari, Christina Kydona, Athina Pyrpasopoulou, Eugenia Avdelidou, Emmanouil Roilidis, Alexandros Garyfallos, Theodoros Dimitroulas

**Affiliations:** 1 Fourth Department of Internal Medicine, Hippokration University Hospital, Medical School, Aristotle University of Thessaloniki, Thessaloniki, Greece; 2 Second Propedeutic Clinic of Internal Medicine, Hippokration University Hospital, Medical School, Aristotle University of Thessaloniki, Thessaloniki, Greece; 3 Third Department of Paediatrics, Hippokration University Hospital, Medical School, Aristotle University of Thessaloniki, Thessaloniki, Greece; 4 Department of Infectious Diseases, Hippokration University Hospital, Medical School, Aristotle University of Thessaloniki, Thessaloniki, Greece; 5 Department of Neurology, Hippokration University Hospital, Thessaloniki, Greece

**Keywords:** Listerial infection of CNS, listeriosis, brain abscess, cerebritis

## Abstract

**Background:**

Listeria monocytogenes is an opportunistic pathogen that causes severe infections of the Central Nervous System, such as meningitis or meningoencephalitis, and brain abscesses. Abscesses account for approximately 1–10% of CNS listerial infections and are observed in 1% of all listerial infections.

**Methods:**

We describe a case of 70-year-old male patient who had several admissions in different hospitals over the last 8 weeks.

**Results:**

He suffered from intermittent fever for over a month, recurrent episodes of headaches, disorientation and other neurological symptoms. His condition was misdiagnosed as giant cell arteritis and initially the patient was started on corticosteroids. MRI of the brain revealed the presence of multiple brain abscesses and the cerebrospinal fluid study confirmed the presence of Listeria Monocytogenes. The patient was started on ampicillin and he completed a 6 weeks’ course of treatment.

**Conclusions:**

This case emphasizes the need to include rare pathogens in the differential diagnosis when possible CNS infections are involved, as well as to show that in many cases some auto-immune diseases are overdiagnosed.

## INTRODUCTION

*Listeria monocytogenes* is a gram-positive facultative intracellular bacterium responsible for severe infections mainly in immunocompromised and diabetic patients, individuals at the extremes of age namely neonates and older adults, pregnant women and occasionally, healthy individuals with no comorbidities.^1,2^ Transmission of Listeria is via the faecal-oral route through the ingestion of contaminated food such as milk, vegetables and meat.

We describe a case of CNS infection with *L. Monocytogenes* presenting as intermittent fever for over a month, recurrent episodes of headaches, disorientation and other neurological symptoms, which were misdiagnosed as possible Giant Cell Arteritis (GCA) and the patient was started on corticosteroids. Our aim is to emphasize the challenges and difficulties of the differential diagnosis in such cases, as well as the overdiagnosis of autoimmune conditions which are treated with corticosteroids.

## CASE REPORT

A 70-year-old male patient was admitted to Internal Medicine Department at the Hippokrateion University Hospital, due to fever up to 39°C for 3 days, accompanied by confusion and disorientation within the last 24 hours. His past medical history included benign prostatic hyperplasia, beta thalassaemia trait and cholecystectomy. A missionary trip to Madagascar was described 8 months prior to the present hospitalization.

The patient had several admissions in different hospitals over the last 8 weeks due to high fever ranging between 38–39°C, associated with recurrent temporal headaches and episodes of transient ischemic attacks presented as hemiparesis, dysarthria, mouth dropping and transient numbness of the right upper limb which were resolving within hours. On the basis of negative blood cultures on several occasions, unresponsiveness to antibiotics, extremely elevated inflammatory markers (Erythrocyte Sedimentation Rate [ESR]=65mm/h, C-reactive protein [CRP]=102mg/L) and a highly suspicious magnetic resonance angiography indicating potential inflammatory stenosis of right vertebral artery and left common carotid artery, the patient was diagnosed with temporal arteritis and treated with IV steroids. Of note, temporal artery biopsy was negative, repetitive computed tomography (CT) scans of the brain did not reveal any abnormality. Following the first course of IV steroids, the patient considerably improved on both clinical and biochemical grounds (ESR=25mm/h, CRP=3,7mg/L). Blood investigations performed over this period are summarized in *Table 1*.

**Table 1.** Blood Investigations of 1st hospitalization.

	**Admission lab. tests**		**Discharge lab. tests**
WBC	17.800(87%NE)	14.600(79%NE)	12.600(76%NE)
HCT(%)	35,3	33	32,3
Hb(g/dl)	11,5	10.5	10,4
PLTs	187.000	251.000	220.000
ESR(mm/h)	65	65	25
CRP(mg/L)	36	102	3,7

Particularly in view of immunology, ANA was weakly positive with titre 1/80 ds-DNA, p- and c- ANCA, rheumatoid factor and anti-CCP antibodies were negative. Serum immunoglobulin and complement tests were normal.

At that point, two weeks after discharge, the patient was admitted to our ward on treatment with prednisolone 60mg daily, methotrexate 20mg/week, clopidogrel 75mg daily and folic acid 5mg/week. On admission he had stable vital signs (B.P=130/90mmHg, HR=92 bmp, SpO2=96%, Temperature=37,5°C), he was fully disoriented and the clinical examination revealed nothing significant. A CT scan of the brain without IV contrast substances showed only a mild asymmetry of the subarachnoid spaces on the frontal lobe. Despite of the high index of clinical suspicion for CNS infection, we were unable to perform a lumbar puncture because of ongoing treatment with clopidogrel. Clopidogrel and methotrexate were discontinued and corticosteroids were reduced to 30mg daily. The initial laboratory tests showed WBC at 13.500 (88% neutrophils), CRP at 80mg/dl (normal values <6 mg/dl), and ESR at 45mm/hr. Differential diagnosis included CNS infection, and/or treatment-resistant autoimmune disease.

After being consulted by the Infections Department, the patient was started on iv intravenous antibiotics and antiviral regimens: Ceftriaxone 2gr bid, Acyclovir 750mg qid, amikacin 1gr, Linezolide 600mg bid, Voriconazole 400mg bid.

**Figure 1.**
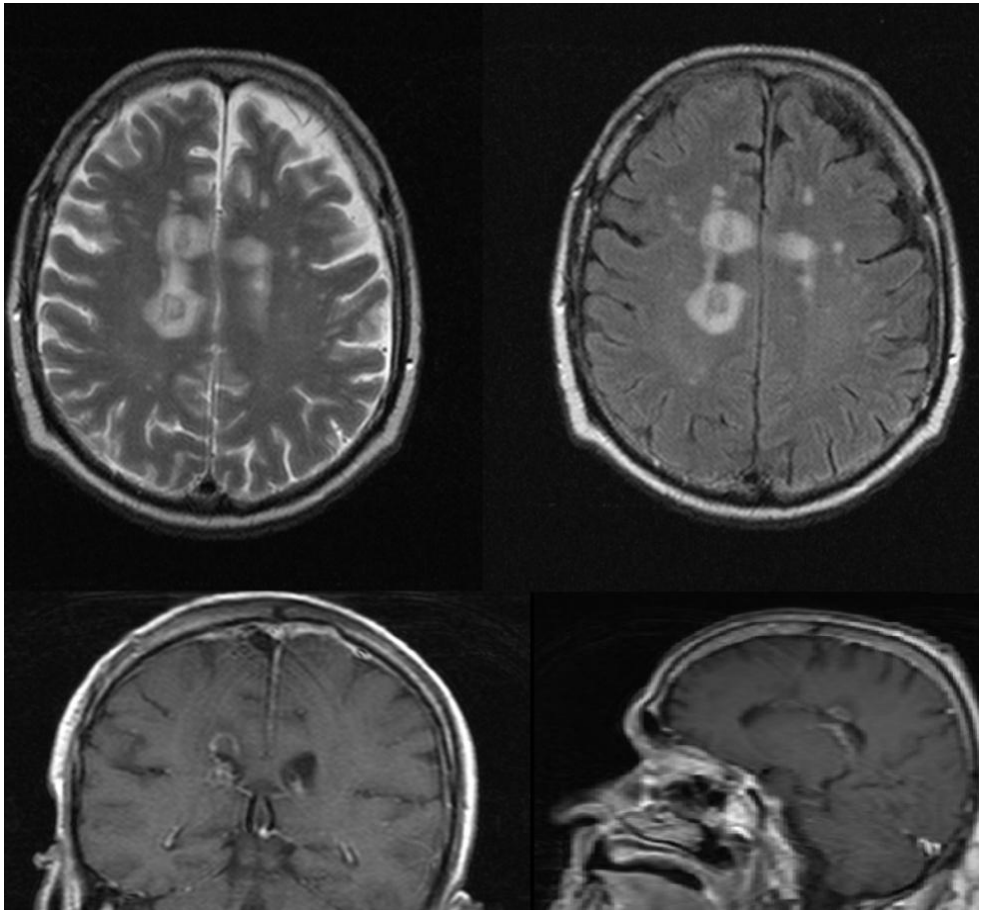
Brain abscesses in axial and sagittal views.

A brain MRI with gadolinium was performed and the radiologist’s official report was as follows: 3 lesions (max diameter 1,3cm) close to the corpus callosum, 2 of them on the right of the midline and 1 on the left, with perifocal cerebral oedema, constriction in the diffusion-weighted sequence and mild ring-shaped enhancement in the right posterior lesion. Two smaller lesions with mild perifocal oedema and peripheral enhancement adjacent to the frontal horn were also reported (*Figure 1*). These findings were strongly indicative of possible abscesses or aspergillus infection. Electroencephalography study was totally normal.

Lumbar puncture was performed and cerebrospinal fluid’s (CSF) chemical profile demonstrated glucose levels at 70mg/dl (normal values 50–80mg/dl or 60–80% of blood glucose), LDH at 34 U/L (normal values 7–30 U/L), and protein at 86mg/dl (normal values 15–45mg/dl). Cellular CSF analysis revealed pleocytosis with WBC count at 60/mm^3^ (normal values up to 5cells/mm^3^), lymphocytes predominance and Gram-positive coccus. A multiplex Real-Time polymerase chain reaction of CSF confirmed infection due to *Listeria monocytogenes*, sensitive to resolution of gastrointestinal symptoms.

**Figure 2.**
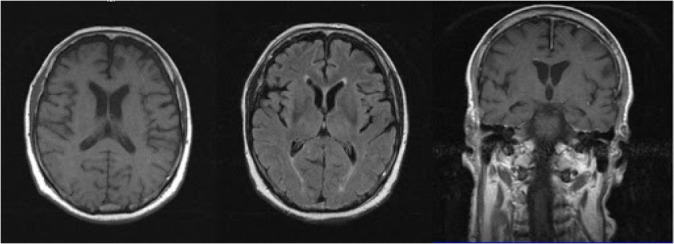
Brain Abscesses after 6 weeks of treatment.

With regards to the overall condition the patient remained afebrile and all of his symptoms subsided after completing 6 weeks of intravenous treatment with ampicillin. A new brain MRI revealed significant improvement (*Figure 2*).

He was discharged on the 50th day with the following instructions: levetiracetam 1,5gr/d, prednisolone 15mg q.d.

**Figure 3.**
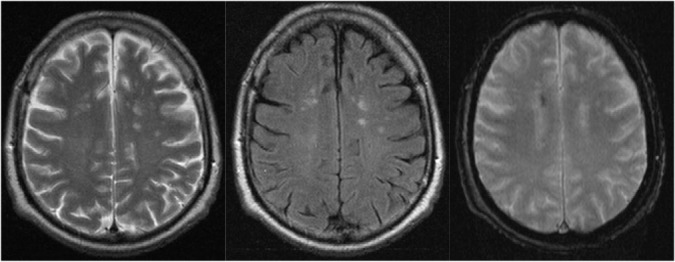
Brain MRI 8 months after treatment.

At the follow-up appointment one month later, his laboratory tests were totally normal, he was in excellent clinical condition and he had started tapering corticosteroids. Eight months after discharge, he is fit, prednisolone treatment has weaned off to 2,5mg on alternate days without any reappearance of fever and headaches, and inflammatory markers are entirely normal (*Figure 3*).

## DISCUSSION

*Listeria monocytogenes* is the third most common cause of bacterial meningitis in North America and Western Europe. Diagnosis should be suspected mainly in immunosuppressed patients. However, studies have shown that abscesses were found in 20% of patients that had no risk factors such as immunosuppression or age. Infection of CNS can be manifested in the form of meningitis or meningoencephalitis, less commonly thromboencephalitis (stem brain involvement) and, quite rarely, in the form of brain abscesses. Brain abscesses are extremely rare as they account for approximately 1–10% of CNS listerial infections and are observed in 1% of all listerial infections.^1,2,3,4^ Because of the rarity of *L. monocytogenes*, it is not always included in the differential diagnosis as the causative agent of brain abscesses. Similarly to our case, most patients with listerial brain abscess are male and over 50 years old.^5,6^ Immunosuppression is a well-recognized risk factor for listerial infection, and patients receiving corticosteroids^2^ are included in this risk group. Corticosteroid therapy increases the susceptibility to listerial infections because of impaired neutrophil function,^7^ and is considered to be the most important predisposing factor in nonpregnant patients. In patients with comorbid diseases, two or more brain abscesses were found.^8^ Blood cultures are usually positive, while 50% of CSF cultures are positive. Approximately 90% of patients with listerial abscesses had symptoms for 2 weeks or less.^5^ The most common symptoms include fever and headache, as well as focal neurological deficits,^5,8^ all of which were present in our patient.

Some features of listerial brain abscess, namely, the presence of bacteraemia, the concomitant meningitis and subcortical abscesses located in thalamus, pons and medulla,^2^ are relatively uncommon in abscesses caused by other bacteria and may contribute to the prompt diagnosis and initiation of appropriate therapy.

Mortality of approximately 30% was reported in patients with comorbidities or receiving immunosuppressant medications and neonates. On the contrary, no deaths were reported in previously healthy patients. Mortality from brain abscesses can be as high as 50%, but is lowered to 40% when appropriate treatment is timely started.

The recommended duration of antimicrobial therapy ranges from 3 to 8 weeks depending on the type of CNS involvement: for example, meningitis vs abscess, as well as patient’s immunological status.^9–11^ According to a recent consensus study, antimicrobial therapy for brain abscess should last 6–8 weeks.^4^ Immunocompromised patients should continue treatment for at least 6–8 weeks with antibiotics until the culture is negative and/or the imaging of the brain is improved.^2,5^

Our patient survived his CNS infection presenting as multiple brain abscesses. We attribute this favourable clinical course to the timely initiation of appropriate antibiotic therapy, despite the delay due to the initial misdiagnosis. He initially presented with minor neurological symptoms that resolved quickly (misdiagnosed as transient ischemic attack) and then demonstrated intermittent fever and headaches, which raised suspicion for temporal arteritis and later for CNS infection. Before the final diagnosis, he was partially treated with oral and intravenous antibiotics, and hence, the disease’s course was prolonged. Furthermore, corticosteroids may have masked the neurological symptoms through decreasing the perifocal oedema surrounding the abscesses. Moreover, he had no history of ingestion of dubiously preserved foods or non-pasteurized dairy products before admission. Thus, a listerial infection was not suspected from the onset of his symptoms. However, our case emphasizes the need for detailed and thorough investigations prior the initiation of corticosteroids in patients with similar symptomatology and no robust evidence of autoimmune disease.

## CONCLUSION

This case emphasizes the need to include rare pathogens in the differential diagnosis when possible CNS infections are involved, as well as to show that in many cases some auto-immune diseases are overdiagnosed.
